# Ninth Annual Meeting of the American Society of Dental Surgeons, Held at Saratoga, August, 1848

**Published:** 1850-10

**Authors:** William Henry Burr

**Affiliations:** Phonographer


### NINTH ANNUAL MEETING OF THE AMERICAN SOCIETY OF DENTAL SURGEONS, HELD AT SARATOGA, AUGUST, 1848.

Reported by William Henry Burr, Phonographer.

First Day, 3½ P. M.

The Society assembled to discuss the subject for the present year, which was "Filling Teeth."

The conversation was opened by Dr. Amos Westcott, who suggested that first in order of the discussion they should take up that part relating to *filing and otherwise separating* the teeth for filling. The suggestion meeting the approbation of the meeting, he proceeded to state his method of practice in this particular. He said (speaking particularly of front teeth) that he used the file pretty freely, and did not now think of but one exception to the use of it, and that was where the teeth approximate so as to come quite together at their points, and not at the gum. In such cases it was his practice to use wedges.

Dr. C. A. Harris said that he had taken some pains to inform himself with regard to a practice which had obtained among many eminent practitioners, that of separating the teeth by means of wedges of wood, or the interposition of some other substance. Previously to the completion of the growth of the body it may be done in most cases with impunity. The operation may often be performed with safety after the age of fifteen or twenty, or even twenty-five or thirty, but as a general rule, it should not be attempted earlier than the fifteenth or later than the twenty-fifth or thirtieth year. When performed earlier or later, it is apt to produce inflammation in the alveolo dental periostal membrane and gums, which, ever after, renders them more liable to disease. A gentleman of the profession, some six years ago, called on me to obtain my opinion with regard to separating the teeth of a lady of a strumous habit of body, twenty-eight years of age, for the purpose of obtaining sufficient room to insert fillings in their approximal surfaces. I suggested that I thought he would be running considerable risk by doing so. He was induced, however, to separate the incisors, cuspidati and bicuspids; in consequence of which a diseased action was set up, which, in four years, resulted in the partial destruction of the alveolar process of the central and lateral incisors, and will, in all probability, occasion the loss of these teeth. I believe the judicious use of the file to be one of the most valuable operations in dental surgery, and in a large majority of the cases, it has been my uniform practice where the patient exceeds twenty-one years of age, to file the approximal surfaces of the teeth preparatory to filling them. Much judgment, however, with regard to the difference of susceptibility of teeth is necessary, and when the proper precautions are used I do not believe as much danger is to be apprehended as some suppose. Even in young persons, where I cannot separate the teeth with wedges, or gum elastic, I have sometimes felt myself called upon to use the file in order to secure a sound solid margin or wall around the orifice of the cavity of the tooth. Also, in cases where the decay has proceeded so far as to break away the enamel from the labio, or palato—approximal angles of the teeth, I believe filing to be the better method of separation.

Dr. E. J. Dunning wished to inquire of Dr. Harris, what were the symptoms which indicated that injury had been done by wedging, and how soon these symptoms appeared?

Dr. Harris. The symptoms are slight elongation of the teeth, inflammation and swelling of the margins and points of the gums which surround them, and ultimately a gradual loosening of the teeth, resulting from destruction of the sockets, depending altogether upon the patient's susceptibility to morbid impression. It is particularly necessary for the dental practitioner to be able to discriminate between these susceptibilities in different individuals. To illustrate my meaning, a scratch upon the hand, will produce in some, but a trifling inconvenience, but in others, it may give rise to extensive inflammation, ulceration, and even gangrene. It is important, therefore, that every dentist who is in the habit of separating teeth with wedges, should be well acquainted with the indications of different constitutional susceptibilities.

Dr. W. H. Dwinelle inferred from the gentleman's remarks, that if he is to draw the teeth apart by elastic bands, he prefers to do it upon persons under the age of twenty years, and generally refrains from doing so on those over that age. In my own experience, I am induced to believe that the misfortune spoken of by the gentleman, was as liable to occur at one age as well as another. In the case of a young girl of thirteen, whose teeth were very irregular, I regulated them in the ordinary way, and the consequence has been elongation and inflammation, and unless I am more fortunate than I expect to be in her case, the destruction of one of the central incisors will ensue. I refer this case however, to a morbid state of the system, the subject is scrofulous. I am not of the opinion that the age has so much to do with the result, but am inclined to think we are unable to anticipate fully and entirely what the result may be in any subject. I regard it as an experiment which will have its occasional failures as well as other operations in our profession.

Dr. Dwinelle heartily endorsed what Dr. Harris had said with regard to the practice of filing teeth. He said, also, that he had made it a point to be particular to polish the surface filled, and not to leave broken and velvet surfaces as so many infinitesimal nucleuses for the collection of foreign matter.

Dr. Harris. In examining this subject, we must do it as pathologists and not as mere mechanics. Previous to the growth of the body, the recuperative energies are more active than at a later period of life. Hence, it is, that an injury of lesion is more readily and perfectly repaired in young subjects than at a period of life subsequently to the age of twenty-five; consequently, there is a greater susceptibility to morbid impressions, provided, the state of the general health is the same. In the case mentioned by Dr. Dwinelle, as in many other cases, it may result in a series of disasters, so that it may not, in some cases, be safe before the age of twenty, to attempt to change the position of a tooth, though, as a general rule, in the treatment of irregularity, the sooner it is instituted, the better.

Dr. E. J. Dunning. I frequently use wedges for the purpose of separating teeth, more especially front teeth, but, I agree with Dr. Westcott, in preferring a thorough use of the file in treating caries upon the lateral approximating surfaces of bicuspids and molares. In treating these cases, I use the file very freely, making wide and permanent separations so shaped, if possible, that they will be cleansed by the constant action of the tongue, cheeks, saliva, &c.

In the treatment of the front teeth, however, the dentist must endeavor to secure, not only the health of the organs, but their beauty; while he remedies the defects caused by disease, he should never forget the importance of these organs to the expression and comeliness of the countenance, and while he is stern and unflinching in his adherence to correct principles of practice, he should not unnecessarily compromise symmetry of form.

There is one circumstance which has an important bearing on this subject of separating teeth which has not yet been mentioned. I refer to the habits of cleanliness of which we may observe the indications in the mouths of our patients. There are some cases in which thorough separations are imperatively demanded, in order to secure stoppings occurring in approximal surfaces or the parts immediately surrounding them from the action of decomposing agents, while in other cases, such precautions are unnecessary, these deposits being removed by the patient once, twice, or thrice, in each day, as the case may require.

Delicacy of constitution—impaired health, acrid secretions from whatever cause or their opposites must enter into the account and assist the dental practitioner in determining the treatment.

Dr. Westcott coincided in Dr. Dunning's remarks, that unless the dentist is governed by the circumstances of the patient, he will make but a poor operator. This saying, that lobelia is good for consumption, and steaming for a fever, may do for quack doctors, but not for us. We do not practice in that way. Let us take a particular case; for example, that of a child, whose teeth are closely crowded, but not uneven. In such a case where I can depend upon the good care of the teeth, my plan particularly for the last two years has been, first to separate the teeth by filing. When the cavities are of large size, I so separate the teeth as that they never will get together again. When the points only of the teeth touch, I have found the filing of the teeth to be bad practice, and if I must get space, I do it by wedges. In the case I have supposed, where the teeth are crowded, I first separate with the file about the thickness of a common visiting card, but do not carry the separation farther up than the apex of the gum. I then use wedges gradually so as to get sufficient room. Then I go through another process of filing in such a manner as to make the edge on the under side, shorter than the outside. This method of filing which can be easily accomplished after the teeth are separated as above described, fulfills two important indications, viz :

1st. It perfectly hides from *front* view, the fillings after they are inserted, although they may be plainly and distinctly seen from behind, when the mouth is open.

2d. The spaces thus left between the teeth, are such as may be with the least effort kept clean. If the proper care be taken in giving the right shape, and in leaving every point perfectly polished, little effort will be required to keep them free from food or other foreign matter. The action of the tongue and the fluids of the mouth will be generally sufficient.

We cannot work as easily upon children as upon adults, and unless we can get sufficient room, my own skill is not sufficient to perform a good operation more than half the time. I never operate but upon two cavities at a time, after completing which, I let them fall back to their natural position, and begin with two more. If I begin upon the lateral cavities, I next take the same on the other side of the mouth, so as to let the irritation subside before commencing another operation about the same spot. If the cavities are small, and the patient one whom I can operate on again soon, I defer the operation till the cavities are more fully developed. Of this, I may speak more fully hereafter, if time affords us an opportunity to discuss the subject of *filling;* with regard to the use of wedges, I accord with Dr. Harris, that there is a liability to chronic inflammation which may sometimes be lasting. Where we find the gum already absorbed with an evident predisposition to absorption with yellowish teeth, if the patient is more than twenty years of age, I conceive it hazardous to wedge, and if cavities are on the lateral surfaces, I should endeavor to get at them in some other way than wedging. If the patient could and would come often enough, so as to prevent the possibility of losing them from neglect, I should certainly leave the cavity until it was justifiable to file. As regards the manner of filing as I have already observed, I make the under portion of the teeth the narrowest. The front teeth should not be disfigured, but kept as near their normal shape as possible. In cases where the irregularity of the teeth is considerable, we are not obliged to file at all, the cavities being sufficiently exposed.

Were the manner of filing the special subject of discussion, I would make some suggestions upon the mode I have adopted in particular cases—here simply observing that by judicious management in performing this operation while the primary object may be to obtain space to enable us to fill the teeth, very much may be done to correct slight or even considerable irregularity of the incisors.

The general idea which I would bring forward, is this—if, for instance, the frontal incisiors are irregular, the right overlapping the left, unless the character of the decay contra-indicates it, I file mainly from the left incisor. The object will be readily understood, when we consider, that the edge left at any point of filing, is more prominent as we advance towards the center of the tooth, and is hence in this way brought more nearly to correspond with the over-lapping tooth, whose contiguous edge was raised at the beginning above the tooth filed.

I have often succeeded in entirely overcoming slight irregularities in this way.

Dr. J. A. Cleveland, remarked, that in the early part of his practice, he was disposed to discard the file. Another dentist, who was then practising in the same community, had been in the habit of using the file freely for several years. I then differed with him, and cautioned him against its too free use. After some few years experience in practising upon the same patients, I discovered that I was in error, in cautioning my elder brother about going too far in the use of the file. Having continued ever since to practice in the same community, and having abundant opportunity to test the effects, I can say, that I have known probably three cases where the free use of the file was successful, to one where a very limited use was so. I can show filings where the bone has been exposed twenty years, and the teeth are sound. A worthy member of this Association, twenty-eight years ago, operated upon many patients at the South, among whom was my brother and one of his neighbors, upon whom I have since attended. I have lately examined their teeth, whose enamel was filed off so long ago, and I found them sound. They are both, however, attentive to their teeth. I now use the file much more freely than formerly. I have never used wedges or any other means. My method is, first, to introduce a very thin file, then I file away what I deem necessary for filling: I then reduce the edges where they come in contact to very small points by filing slightly from each side above and below, so as to bring the approximal points on as small spaces as possible. I file bicuspids and molars more than front teeth. I concur with Dr. Westcott, with regard to filing under; most of my fillings are put on the under side. I have used the file in the manner described for fifteen years.

Dr. J. H. Foster. My treatment of the front teeth differs very much in respect to the mode of separation from that which I pursue with bicuspids and molars. I occasionally use wedges in separating the latter cases, but more frequently make free and wide spaces with a chiselled-shaped instrument, and then with the file complete the operation for making the space between the teeth level and regular, leaving it in form similar to the letter V. I use the file between the front and lateral incisors and the cuspidati, only so far as to secure the separation which they require after the operation of filling is performed, except in those cases where the parieties of the cavities are so thin and weak as to require that more should be removed. So much injury has been done by the indiscriminate and reckless use of the file, for which the poor persecuted instrument has borne the obloquy which should have attached to the hand that used it, and yet it is powerful for good as well as evil, if treated kindly, that I have been more cautious in handling the file than any other instrument. With respect to wedging, I prefer this course of treatment, and as a general rule am governed by it in separating the front teeth. I use India rubber in preference to wood, because I think it less irritating. I have three or four different thicknesses, which I insert successively in all cases in which it is possible. Wait until the inflammation consequent upon the pressure has entirely passed away, before performing the operation of filling; the severe inflammation spoken of has never come under my notice in any case, and must be attributed, I think, to too great a degree of pressure upon the teeth in the commencement of this treatment.

Dr. Robert Nelson. If the patient be young—say under twenty—and the cavity small, I press the teeth apart by means of fine linen or cotton cloth, drawn between them—increasing the folds as circumstances may direct.

Should the decay have progressed so far as to make it necessary to remove a portion of the tooth, in order to give form and strength to the sides of the cavity, but not sufficient to give the requisite space for filling, I remove some of the soft bone, and fill the cavity and intermediate space with cotton: the cotton, by absorbing the fluids of the mouth, swells and presses the teeth apart with a gentle, though irresistible force.

I have used various articles for this purpose, but find none to possess the advantages of cotton; it is not only easily applied and retained in the cavity, but it also, while it separates the teeth, presses the *gum* from the margin of the cavity—an office of the greatest importance.

I may here add, that although I have been in the habit of pressing teeth apart for the last fifteen years, yet I have never seen the slightest evil consequences resulting therefrom.

For the back teeth, my *general* practice is to separate them with a chisel, or file; and I feel assured from my own experience in a practice of many years, and from observation on the practice of others, that these instruments may be used with perfect safety by the skillful operator; indeed, I have always observed that those who have been most successful in their practice, have used them with the greatest freedom.

When teeth approximate at the points, the tendency of the wedge is towards the gum, which produces the irritation described by Dr. Harris. It is my practice in such cases, to pass a thin file between, which gives room for the wedge, and prevents the crowding towards the gum.

Dr. Westcott thought that the difference in the kind of wedges used, arose from one's making a right use of one kind, and seeing a bad use of the other. He had several years used rubber principally, but now prepared fine wood; perhaps if he had adopted the assorted India rubber at first, he might have approved of it better. He liked wood also, because it will retain the teeth in their places. By shaping the plug like an hour-glass, it can be worn several days till the soreness gets out, which he was unable to do with gum elastic.

Dr. Harris said he had tested the relative merits of both wood and gum elastic, having used them indiscriminately for a number of years. To separate teeth, a certain amount of inflammation is necessarily excited, by the pressure of the organs against the alveolo-dental periosteum, and the greater the pressure the more active, as a matter of course, will be the inflammation. When the pressure is exerted very slowly, the pain will be slight, causing the patient but little inconvenience. But, as I have before stated, a certain amount of inflammation is necessarily produced, and as the alveolo-dental periosteum becomes inflamed, a chemical fluid is poured out, which breaks down the surface of the alveolar wall, against which the tooth is pressed, and it is partly in this way, and partly by the yielding of the alveolus that the tooth is moved. It is true, the irritation may, and often is, very greatly increased by the pressure of the interposed substance against the point of the gum.

Dr. Harris stated that he had seen teeth which had been filed thirty-five years, that were still sound, yet many teeth thus filed, may have decayed on account of the greater susceptibility of the organs to the action of the causes that produce the structural alteration. This should always be considered, as well as the patient's habits of cleanliness, with regard to the teeth. Some teeth may be able to resist the action of the usual causes of caries, throughout the whole period of life, if the filed surfaces be kept thoroughly and constantly clean, but teeth of a soft texture will ultimately be decomposed. But as I have elsewhere given my views upon the manner of performing this operation, as well as upon its utility, it is unnecessary to say more upon the subject.

Dr. J. B. Rich. In the early part of my practice, I was informed of Dr. Parmly's method of separating the teeth by means of wedges of wood. I availed myself of this method whenever I met with an opportunity to do so. The result was by no means satisfactory. In several cases, so great was the inflammation superinduced, that the teeth lost their vitality, consequently I abandoned the use of wood for that purpose.

(TO BE CONTINUED.)

